# Hon. Wilson Shannon Bissell

**Published:** 1903-11

**Authors:** 


Hon. Wilson Shannon Bissell, chancellor of the University of Buffalo, died at his residence in this city, October 6, 1903, aged 55 years. Mr. Bissell had been prominent in the affairs of Buffalo for many years and ranked as one of its ablest lawyers as well as one of its most respected citizens. He served as postmaster general in the second cabinet of President Cleveland, and belonged to various local organisations, including Buffalo Historical Society, all of which adopted memorial minutes.

The faculty of the medical department of Buffalo University adopted the following:

The faculty of the medical department of the University of Buffalo wish to express their profound sorrow for the death of the Honorable Wilson Shannon Bissell. Serving as he did for many years on the council of the university, as member, vice-chancellor and later as chancellor, he always had shown a hearty interest in its welfare and had ever been ready to forward its interests in every way in his power.

As a model citizen, a brilliant lawyer, a devoted public servant and best of all, as a noble Christian man, he will long be remembered; but his effective work for the University of Buffalo must be put among his good deeds not to be forgotten. While his death will be deeply felt by our citizens in general and by the nation at large, we feel that to us his loss comes with especial force, as at this particular time the university greatly needs his sound advice and accumulative wisdom and will miss them in proportion; therefore, be it

*Resolved,* That this expression of our appreciation of Mr. Bissell and of our strong sense of loss be spread upon the minutes and a copy be sent to his family. Signed, Matthew D. Mann, Dean; Charles Cary, Roswell Park, Charles G. Stockton, John Parmenter, Herbert M. Hill and Eli H. Long.

Mr. Bissell's remains were incinerated at the Buffalo crematory, and the ashes were buried in the family lot at Forest Lawn.

